# Implication of Caspase-3 as a Common Therapeutic Target for Multineurodegenerative Disorders and Its Inhibition Using Nonpeptidyl Natural Compounds

**DOI:** 10.1155/2015/379817

**Published:** 2015-05-04

**Authors:** Saif Khan, Khurshid Ahmad, Eyad M. A. Alshammari, Mohd Adnan, Mohd Hassan Baig, Mohtashim Lohani, Pallavi Somvanshi, Shafiul Haque

**Affiliations:** ^1^Department of Clinical Nutrition, College of Applied Medical Sciences, University of Ha'il, Ha'il 2440, Saudi Arabia; ^2^Department of Biosciences, Integral University, Lucknow, Uttar Pradesh 226026, India; ^3^School of Biotechnology, Yeungnam University, Gyeongsan 712749, Republic of Korea; ^4^Department of Biotechnology, TERI University, New Delhi 110070, India; ^5^Research and Scientific Studies Unit, College of Nursing & Allied Health Sciences, Jazan University, Jazan 45142, Saudi Arabia; ^6^Department of Biosciences, Jamia Millia Islamia, New Delhi 110025, India

## Abstract

Caspase-3 has been identified as a key mediator of neuronal apoptosis. The present study identifies caspase-3 as a common player involved in the regulation of multineurodegenerative disorders, namely, Alzheimer's disease (AD), Parkinson's disease (PD), Huntington's disease (HD), and amyotrophic lateral sclerosis (ALS). The protein interaction network prepared using STRING database provides a strong evidence of caspase-3 interactions with the metabolic cascade of the said multineurodegenerative disorders, thus characterizing it as a potential therapeutic target for multiple neurodegenerative disorders. *In silico* molecular docking of selected nonpeptidyl natural compounds against caspase-3 exposed potent leads against this common therapeutic target. Rosmarinic acid and curcumin proved to be the most promising ligands (leads) mimicking the inhibitory action of peptidyl inhibitors with the highest Gold fitness scores 57.38 and 53.51, respectively. These results were in close agreement with the fitness score predicted using X-score, a consensus based scoring function to calculate the binding affinity. Nonpeptidyl inhibitors of caspase-3 identified in the present study expeditiously mimic the inhibitory action of the previously identified peptidyl inhibitors. Since, nonpeptidyl inhibitors are preferred drug candidates, hence, discovery of natural compounds as nonpeptidyl inhibitors is a significant transition towards feasible drug development for neurodegenerative disorders.

## 1. Introduction

**Figure 1:**
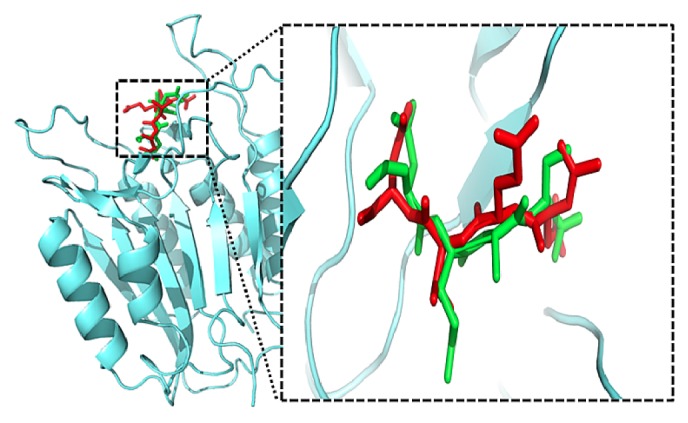
The binding orientation of original (red) and redocked (green) confirmation of peptidyl ligand with the receptor caspase-3.

Neurodegenerative disorders are characterized by progressive loss of structure or function of neurons leading to neuronal death and are frequently hereditary. Some of these common neurodegenerative disorders include Alzheimer's disease (AD), Parkinson's disease (PD), Huntington's diseases (HD), and amyotrophic lateral sclerosis (ALS) as well as few others. In pathological terms, these diseases share a common feature, that is, the selective loss of a particular subset of neurons for unknown reasons. Neurodegenerative disorders, such as, Alzheimer's and Parkinson's disease, account for a significant and increasing proportion of morbidity and mortality in the developed world [1, 2]. Apoptosis has recently been implicated as a possible mechanism for neuronal death in neurodegenerative diseases (AD, PD, HD, and ALS) [3] and has been observed in a large number of other pathological conditions, including ischemia-reperfusion injury (stroke and myocardial infarction), and cardiomyopathy, sepsis, type I diabetes, and allograft rejection [4, 5]. Caspases form a unique class of cysteine aspartate-specific proteases according to their substrate specificities and biological functions [6, 7]. Caspases are proteolytic in nature and key executioners of apoptosis [8]. Excessive neuronal apoptosis leads to a variety of diseases, such as, stroke, AD, HD, and PD [9, 10]. The caspase family consists of cysteine proteases that cleave the peptide bond next to an asparatic acid in its substrates. They are classified as inflammatory and apoptotic caspases based upon their function and prodomain structure. Caspases can be classified into two broad categories, first, initiator caspases (caspase-2, caspase-8, caspase-9, and caspase-10) and, second, effector caspases (caspase-3, caspase-6, and caspase-7). In general, the initiator caspases mostly act in early phases of a proteolytic cascade, whereas effector caspases act downstream and are associated with the cleavage of specific cellular proteins [11]. Under* in vitro* conditions, it has been found that caspase-3 prefers the peptide sequence DEVDG (Asp-Glu-Val-Asp-Gly) along with cleavage taking place on the carboxy side of the second aspartic acid residue (between D and G) [12]. The protein/peptide substrate chain fits in the binding site with the scissile bond positioned close to the catalytic residues (refer to Figure 1 of Stennicke and Salvesen's study [12]). The amide groups of Gly238 and Cys285 donate H-bonds to the carbonyl oxygen, thus polarizing the carbonyl group of the scissile bond [12]. The carbonyl carbon is now electrophilic and susceptible to attack by the nucleophilic thiol of the catalytic Cys285. Prior to or during the nucleophilic attack on the carbonyl carbon, the thiol group of Cys285 donates its proton to His237, which then can act as the catalytic acid by protonating the *α*-amino group of the preceding amino acid. During deacylation of the enzyme His237 will help to polarize the water molecule required for completing the hydrolysis by forming the second tetrahedral intermediate. The inhibitors of this protein are useful in the treatment of cardiomyopathy and neurodegenerative diseases [13]. Caspase-3 is of particular interest among all caspases since it is involved in progression of AD [14] and also in PD, ALS, and HTT disease [15, 16].

ALS is the degeneration of upper (motor cortical) and lower (brainstem and spinal) motor neurons finally ensuing into progressive paralysis. High caspase-3 activity has been detected in humans with ALS [17, 18]. Caspase-3 has been found to cleave excitatory amino-acid transporter-2 (EAAT2) at a specific cytosolic c-terminal site resulting in the selective inhibition of this transporter hence playing a crucial role in the pathogenesis of ALS [19]. Neurons expressing caspase-3 were found to be much more sensitive to the neuronal loss than those that do not express the protein in PD patients [20]. Caspase-3 showed increased immunoreactivity in melanized neurons of the PD nigra compared with controls [21]. Caspase-3 was found to be upregulated in HD patients. Caspase-1 was also found to activate caspase-3 [22, 23]. Sequential proteolysis by caspase-3 and calpain have been found to regulate huntingtin function at membranes and resulted in the generation of N-terminal mutant fragments that aggregate and cause cellular dysfunction in HD patients [24].

Till now, four proteins have been recognized as critical etiological factors for AD, namely, apolipoprotein E, presenilin-1 (PSEN-1), presenilin-2 (PSEN-2), and amyloid precursor protein (APP). Various caspase-3 cleavage sites have been detected and mapped for three of above mentioned proteins [15]. Activated caspase-3 has been detected in neurons of AD brains along with a colocalization of Neurofibrillary Tangles (NFTs) and senile plaques suggesting the role of caspase-3 in synaptic degeneration during the AD progression [15]. Caspase-3 has also been shown to cleave APP, generating A*β*-containing peptides [25]. Activation of caspases and apoptosis of dopaminergic neurons have been implicated in the pathogenesis of PD [26]. PARK2, SNCA, and MAPT have been identified as PD associated proteins playing potential role in apoptosis. Activated caspase-3 has been shown in the substantia nigra of mesencephalon (midbrain) of patients affected with PD [20, 21]. More recently, caspase-1 and caspase-3 activities have been found to be elevated in HD brain and in animal models of the disease and both enzymes have been shown to cleave normal and mutated huntingtin (HTT) under* in vitro* conditions [27]. Caspase-3 activation may have real pathological consequences in mouse model of ALS [28]. Most ALS cases are sporadic, but 5–10% of cases are familial, and among these 20% of cases show mutations within the SOD1 gene (OMIM number 105400). Notably, SOD1 is also responsible for 1.5% of sALS, suggesting a possible role of this protein in both forms of the disease [28, 29]. Earlier studies performed in transgenic mSOD1 mice have shown that activated caspase-3 and its resultant *β*-actin cleavage fragments have been present in apoptotic neurons in the anterior horn of the spinal cord, while such neurons are limited in number but their paucity should not challenge the significance of apoptosis in the overall mSOD1-related neurodegeneration [30].

Since caspase-3 is a proteolytic enzyme, hence, most of the known potent inhibitors of caspase-3 are basically peptidyl in nature. Peptide aldehydes having electron-deficient carbonyls that interact are the most common caspase-3 inhibitors, for example, Z-DEVD fluoromethyl ketone (PDB ID: 2CJX), tetrapeptide aldehyde inhibitor AC-DEVD-CHO (PDB ID: 2H5I), and Ac-DEVD-CMK (PDB ID: 4JRO). The X-linked inhibitor of apoptosis and the neuronal inhibitor of apoptosis are proteins in neurons that directly inhibit caspase-3 activity and protect neurons from ischemic injury [31]. Since peptidyl inhibitors are not preferred drug candidates, so there is a need to search nonpeptidyl inhibitors of caspase-3 which can mimic the inhibitory action of peptidyl inhibitors. In this study, we have explored the binding of some nonpeptidyl natural compounds against caspase-3. This study is a novel attempt to explain the mimicking of inhibitory action of peptidyl inhibitors by nonpeptidyl natural compounds. Gold (Genetic Optimization for Ligand Docking) fitness score as well as X-scores have been used to confirm the binding efficacies of the selected nonpeptidyl inhibitors.

## 2. Materials and Methods

### 2.1. Caspase-3 Interaction Network Discovery

The potential interactions between the proteins (CASP 3, SOD1, PSEN 1, HTT, and PARK 2) were examined using the software program called Search Tool for the Retrieval of Interacting Genes (STRING, Version 9.0). The STRING is a database of known and predicted protein interactions, which includes indirect (functional) and direct (physical) associations, and those are taken from four main sources, that is, previous knowledge, high-throughput experiments, genomic context, and coexpression studies [26]. The STRING program gives exclusively wide-ranging coverage and comfort of access to both experimental and predicted protein interaction information. The protein interactions available in STRING database are delivered with a confidence score. Additional information, such as, pertinent protein domains and 3D structures, is provided within a stable and consistent identifier space. The current STRING program (Version 9.0) covers more than 1100 fully sequenced organisms [32]. However, some recent publications mention the updates in STRING program (as version 9.1) and describe the improvements over the previous versions, which comprises the automated mining of the scientific data for protein interaction information, addition of full-text research articles, redesigning the algorithm for transferring interactions from one model organism to the another one, and relevant statistical information on any functional enrichment detected in networks [33].

### 2.2. Molecular Docking

Molecular docking was performed using GOLD V. 5.0 [34, 35] to decipher the binding affinity and mode of interaction of the selected natural compounds against caspase-3. GOLD is a program for calculating the docking modes of small molecules in protein binding sites and it uses a genetic algorithm methodology with the advantage that it allows both unconstrained ligand flexibility and partial flexibility of the binding pocket. Docking annealing parameters for van der Waals and hydrogen bonding were set at 5.0 and 2.5, respectively. The following parameters were used for genetic algorithm: population size 100, selection pressure 1.2, number of operations 1,00,000, number of islands 5, niche size 2, migrate 10, mutate 100, and cross-over 100. The best ranked “poses” were further analyzed using X-score [36, 37], which is a consensus scoring function to calculate the binding affinity.

### 2.3. Retrieval and Preprocessing of Caspase-3

The crystal structure of caspase-3 was retrieved from the protein databank (PDB), PDB ID: 1PAU. The structure was then subjected to energy minimization using Conjugate Gradient method for 2000 steps and root mean square (RMS) gradient of 0.1 Å after applying the CharMM force field [38] using Discovery Studio (DS) 2.5 software program.

### 2.4. Validation of Size, Grid Center, and Binding Conformation

Prior to performing the molecular docking studies, validation was done, in which the peptide inhibitor present within the active site of the crystal structure of caspase-3 was extracted. This inhibitor was subjected to redock within the active site of caspase-3 using GOLD [34, 35]. The orientation of the crystal and redocked confirmation of the inhibitor was compared with crystal structure (PDB ID: 1PAU) obtained from the protein data bank.

### 2.5. Retrieval and Preprocessing of Natural Compounds

The 3D structures of the selected natural compounds with known antineurodegenerative properties were retrieved from PubChem compounds database. These compounds included bilobalide (PubChem ID: 12308750), quercetin (PubChem ID: 5280343), EGCG (PubChem ID: 65064), resveratrol (PubChem ID: 445154), curcumin (PubChem ID: 969516), huperzine A (PubChem ID: 5912039), rosmarinic acid (PubChem ID: 5281792), luteolin (PubChem ID: 5280445), apigenin (PubChem ID: 5280443), and berberine (PubChem ID: 2353).

## 3. Results

**Figure 2:**
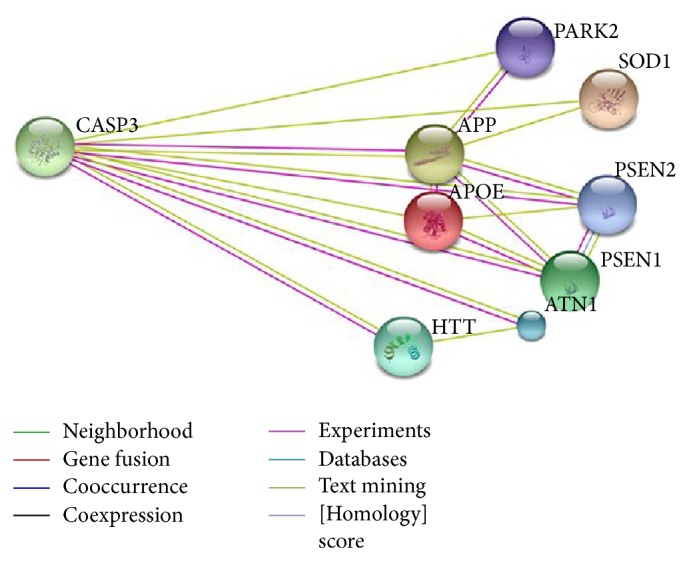
Protein interaction network of caspase-3 with PSEN1, HTT, PARK2, and SOD1 proteins using STRING (http://string-db.org/).

Protein interaction discovery was performed using STRING database. The interaction network of caspase-3 with the metabolic cascade of multiple neurodegenerative disorders (AD, PD, ALS, and HD) is shown in Figure 2. STRING provides a comprehensive coverage involving access to both experimental and predicted interaction information. The interactions available in STRING software program are provided with confidence score along with some additional information (protein domains and 3D structures involved in interaction). The prediction techniques employed for the determination of possible interaction involve databases and text mining, cooccurrence, gene fusion, neighborhood, experiments, and coexpression. The required confidence score (cutoff score) was set to be 0.400. STRING predicted interaction confidence scores are given in Table 1. The interaction between input proteins is color coded representing the type of interactions (Figure 2). Evidences suggesting a functional link between CASP3 and other input proteins are determined by text mining and are represented by green color; however, experimental/biochemical data is represented by pink color interaction.

The binding potential of the selected natural compounds with caspase-3 was determined by performing* in silico* binding of these compounds with the target (i.e., caspase-3). PubChem database was screened for natural compounds possessing antineurodegenerative potential. Nonpeptidyl natural compounds selected for molecular docking studies are listed in Table 2.* In silico* redocked inhibitor was found to interact with the same amino acids of the active site as in the original crystal structure (Figure 1). The root mean squared deviations (RMSD) of all atoms between these two conformations (redocked and original crystal structure from protein data bank) were found to be 1.87 Å only, suggesting that the protocol set for molecular docking is fairly efficient and faithfully reproduces the crystallographic complex with a high degree of similarity. The orientation of the crystallized and* in silico* redocked peptide inhibitor is shown in Figure 1 and the types of interactions are reported in Table 3.

**Figure 3:**
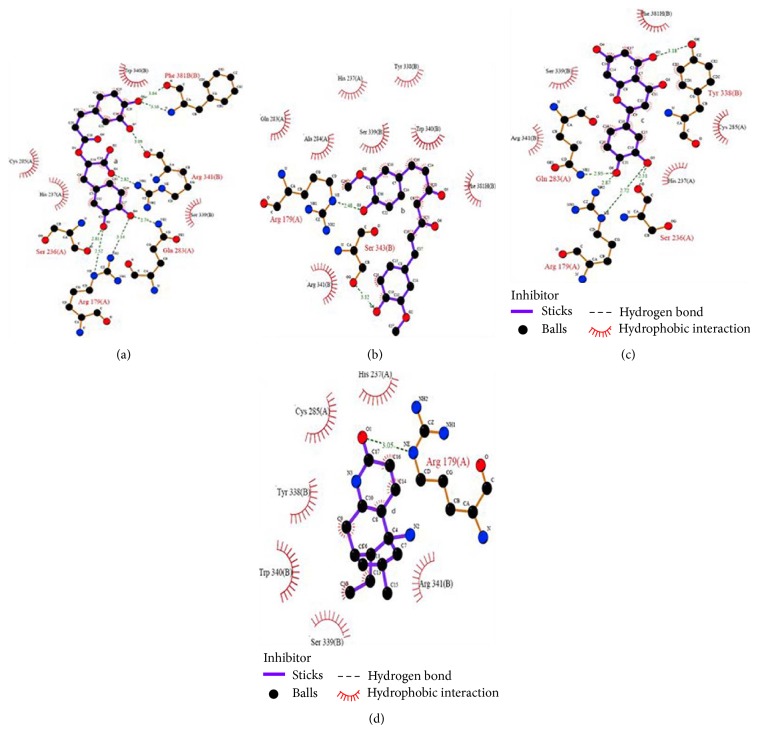
Visual representation of the interaction of (a) rosmarinic acid; (b) curcumin; (c) luteolin; and (d) huperzine A with different amino acids within the active site of caspase-3.

Molecular docking simulations were used to investigate the possible binding modes of the selected natural compounds within the active site of caspase-3. Several plausible docked structures were detected. Those docked structures were scored, using two different scoring functions: GOLD and X-score. The derivatives were finally ranked on the basis of results obtained from GOLD and X-score. It was found that rosmarinic acid, curcumin, luteolin, and huperzine A were capable of binding within the binding site of caspase-3 with high affinity (as compared to other compounds considered in this study). The docked structures are shown in Figures 3(a), 3(b), 3(c), and 3(d).

**Figure 4:**
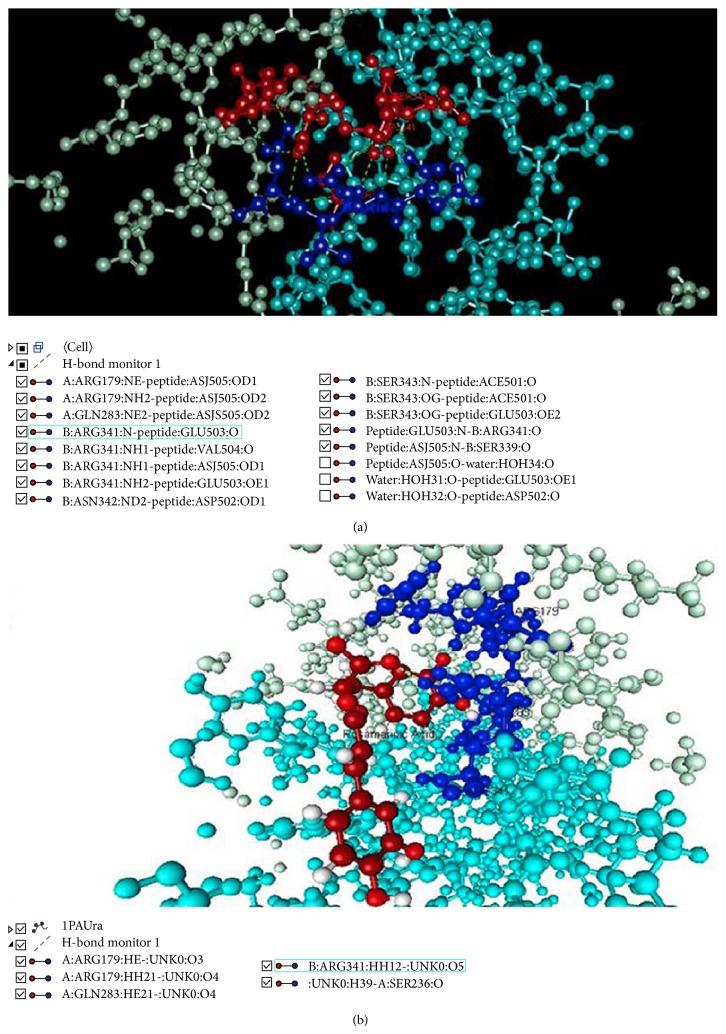
Hydrogen bonding interaction of peptidyl and nonpeptidyl inhibitors with caspase-3. (a) Hydrogen bonding interaction of peptidyl inhibitor with caspase-3. (b) Common hydrogen bonding interaction of rosmarinic acid with caspase-3. (Ligand/inhibitor: red; amino acids of caspase-3 interacting with inhibitors via hydrogen bonding: blue; Unk0: rosmarinic acid.)

Caspase-3 docked with a tetrapeptide aldehyde inhibitor AC-DEVD-CHO (PDB ID: 1PAU) along with possible hydrogen bonding interactions (between tetrapeptide and caspase-3) are shown in Figures 1, 4(a), and 4(b). It can be observed that most of the hydrogen bond interactions of the peptidyl inhibitor resemble the hydrogen bonding of rosmarinic acid with caspase-3 (Figure 1, Table 3, and Figures 4(a) and 4(b)). The similar hydrophobic interaction of rosmarinic acid with Cys-285 and other amino acids further stabilized the bounded conformation (Table 3).

## 4. Discussion

Currently,* in silico* approaches are routinely used in modern drug design and discovery programs to understand the gene pathway network as well as drug receptor interaction. It is evident from the previous literature that these computational approaches can intensely support and help the design of novel, more potent inhibitors by deciphering the mechanism of drug receptor interaction. The major objective of this study is to identify common therapeutic target(s) involved in multineurodegenerative disorders and to detect promising leads against them. Protein interaction discovery performed using STRING database revealed that caspase-3 has significant interaction with the core proteins (APP, HTT, PSEN2, PARK2, and SOD1; refer to Table 1) involved in the molecular cascade of multiple neurodegenerative disorders, like AD, PD, ALS, and HD. This identifies caspase-3 as potent common therapeutic target for multiple neurodegenerative disorders.

Molecular docking is considered to be an important tool to investigate the mode of interaction of ligand with the target and to elucidate the underlying binding mechanism. Gold docking platform is a known academic standard in molecular docking studies. Redocking of the peptide inhibitor (present within the active site of retrieved crystal structure of caspase-3) was performed to acquire and validate the size, center of the coordinate grid, and ligand binding conformation in the binding pocket. Among all the natural compounds tested in this study, rosmarinic acid was found to be the most effective caspase-3 inhibitor (gold fitness score of 57.38). Rosmarinic acid is a polyphenol antioxidant carboxylic acid existing in many herbs of Lamiaceae family and possesses several biological activities, which comprises antibacterial, antioxidant, antiviral, anti-inflammatory, anticancer, and neuroprotective effects [39, 40]. Rosmarinic acid has been reported to obstruct apoptotic pathways by impeding ROS formation, DNA fragmentation, and caspase-3 activation [41].

Several preclinical studies on curcumin suggest that it may be useful for the treatment of several diseases, like cancer, cystic fibrosis, and inflammatory diseases as well as neural disorders [42]. Previous reports also suggest that curcumin can also inhibit aluminum-induced oxidative stress and mitochondrial dysfunction in rat brain [43]. In this study, curcumin was found to be another active caspase-3 inhibitor. We found that curcumin bounds with gold fitness score of 53.51 against caspase-3. This suggests that curcumin is another active compound that could be further investigated and can be exploited as a caspase-3 inhibitor in near future. Caspase-3 was also found to be inhibited by luteolin with gold fitness score of 51.24. Luteolin is a yellow crystalline flavonoid found in Pinophyta, Bryophyta, Pteridophyta, and Magnoliophyta. It has already been reported that luteolin possesses a wide range of pharmacological properties including anticancer and neuroprotective activities [44]. Huperzine A, a naturally occurring sesquiterpene alkaloid found in the firmoss* Huperzia serrata* [45]. Huperzine A was also found to be very active as a caspase-3 inhibitor (gold fitness score of 51.13). Earlier studies on huperzine A also reported that this compound has a potential for inhibiting apoptotic factors, such as caspase-3 along with regulating the expression and secretion of nerve growth factors [46].

Most of the known inhibitors of caspase-3 are peptides, unattractive candidates for drug development. The catalytic site of caspase-3 involves the sulfhydryl group of Cys-285 (A chain) and the imidazole ring of His-237 (A chain). His-237 stabilizes the carbonyl group of the key aspartate residue, while Cys-285 attacks and ultimately cleaves the peptide bond. Cys-285 (B chain) and Gly-238 (B chain) also act to stabilize the tetrahedral transition state of the substrate-enzyme complex through hydrogen bonding [12].

Rosmarinic acid proved to be the finest nonpeptidyl inhibitor (among all the compounds included in this study) mimicking the binding of the peptide substrate/inhibitor with caspase-3. List of similar hydrogen bonding interactions is shown in Table 3. Most of the hydrogen bond interactions of the peptidyl inhibitor resemble the hydrogen bonding of rosmarinic acid with caspase-3. The hydrophobic interaction of rosmarinic acid with Cys-285 and other amino acids further stabilize the bounded conformation (Table 3). Rosmarinic acid also shows maximum number of hydrogen bonding interactions with caspase-3 as compared to other inhibitors (Table 3 and Figure 4). Since the hydrogen bonding and hydrophobic interactions of rosmarinic acid closely resemble the interaction and orientation of peptidyl inhibitor, there exists a high probability that an almost similar and stable tetrahedral transition state may result thereby stabilizing the interaction of rosmarinic acid with caspase-3.

These results suggest that nonpeptidyl inhibitor, rosmarinic acid, is the most efficient inhibitor of caspase-3 in terms of amino acid interaction and gold fitness score. The results of GOLD Platform were further validated using another binding fitness scoring tool X-score (a consensus based scoring function to calculate the binding affinity). The results of gold fitness were found to be in close agreement with the fitness score predicted through X-score (Table 1).

## 5. Conclusions

In this study, we have used* in silico* approaches to explore the vital role of caspase-3 in multineurodegenerative disorders thereby establishing it as common potent drug target for the said neurodegenerative disorders. Also, we tested the binding orientations of several plant-derived nonpeptidyl natural inhibitors against this proteolytic enzyme. Since most of the known inhibitors of caspase-3 are peptidyl in nature, not preferred drug candidates, thus the discovery of nonpeptidyl inhibitors of caspase-3 is a significant step towards designing potent drug candidate against neurodegenerative disorders. The results obtained from this study would be useful in both understanding the inhibitory mode of the plant derived natural compounds and comparing their inhibitory actions to peptidyl inhibitors.

## Supplementary Material

Caspase 3 has been targeted by several researchers to combat multi-neurodegenerative (viz., Alzheimer's disease, Parkinson's disease, Huntington's diseases, and Amyotrophic lateral sclerosis) disorders. Several peptidyl inhibitors of caspase-3 have been identified. Owing to inherent difficulty in the handling and drug development from peptides, peptidyl inhibitors are not the preferred leads. Peptide based drugs result in higher drug development and handling cost. Moreover the efficiency of peptide may be compromised if the storage environment somehow deviates from the prescribed conditions. We have identified potent natural non-peptidyl inhibitors of caspase-3, effectively mimicking the interactions and binding affinity of peptidyl inhibitors.

## Figures and Tables

**Table 1:** STRING predicted interaction confidence scores.

Protein I	Protein II	Interaction confidence score
CASP3	Amyloid precursor protein (APP)	0.764
CASP3	Presenilin-2 (PSEN2)	0.0745
CASP3	Huntingtin (HTT)	0.782
CASP3	Superoxide dismutase (SOD1)	0.506
CASP3	Alpha-synuclein (PARK2)	0.0430

**Table 2:** Binding affinity of nonpeptidyl natural compounds against caspase-3.

Compounds	Gold fitness score	X-score
Rosmarinic acid	57.38	−8.93
Curcumin	53.51	−8.14
Luteolin	51.24	−8.51
Huperzine A	51.13	−7.97
Quercetin	44.33	−6.98
Resveratrol	42.65	−6.53
Bilobalide	43.74	−6.56
EGCG	47.15	−7.02
Apigenin	41.32	−6.97
Berberine	40.56	−5.48
Chitosan	37.67	−5.97

**Table 3:** List of hydrogen bonding and hydrophobic interactions of nonpeptidyl and peptidyl inhibitors with the amino acids of caspase-3.

Inhibitors	Residues involved	
	Hydrogen bond	Hydrophobic interaction
Nonpeptidyl		
Rosmarinic acid	***ARG179***, ***SER236***, ***GlN283***, ***ARG341***, PHE381	***HIS237***, ***CYS285***, SER339, ***TRP340***, and ***ARG341***
Curcumin	***ARG179***, and ***SER343***	***HIS237***, GLN283, ALA284, TYR338, SER339, ***TRP340***, ARG341, and PHE381
Luteolin	***ARG179***, ***SER236***, ***GLN283***, and TYR338	***HIS237***, ***CYS285***, TYR338, SER339, ***ARG341***, and PHE381
Huperzine A	***ARG179***	***HIS237***, ***CYS285***, TYR338, SER339, ***TRP340***, and ***ARG341***
Peptidyl		
Tetrapeptide aldehyde AC-DEVD-CHO	***ARG179***, ***SER236***, ***GlN283***, ***ARG341***, ***SER343***, and ***SER339***	***HIS237***, ***CYS285***, ***TRP340***, ***ARG341***, and ***SER343***

Bold and italic amino acids represent similar interaction as observed for peptidyl inhibitor.
