# Supplementary figures and images for: Implication of Caspase-3 as a Common Therapeutic Target for Multineurodegenerative Disorders and Its Inhibition Using Nonpeptidyl Natural Compounds
Source: Biomed Res Int. 2015 May 4;2015:379817. doi: 10.1155/2015/379817 (PMC4434175; doi:10.1155/2015/379817)

## Graphical Abstract

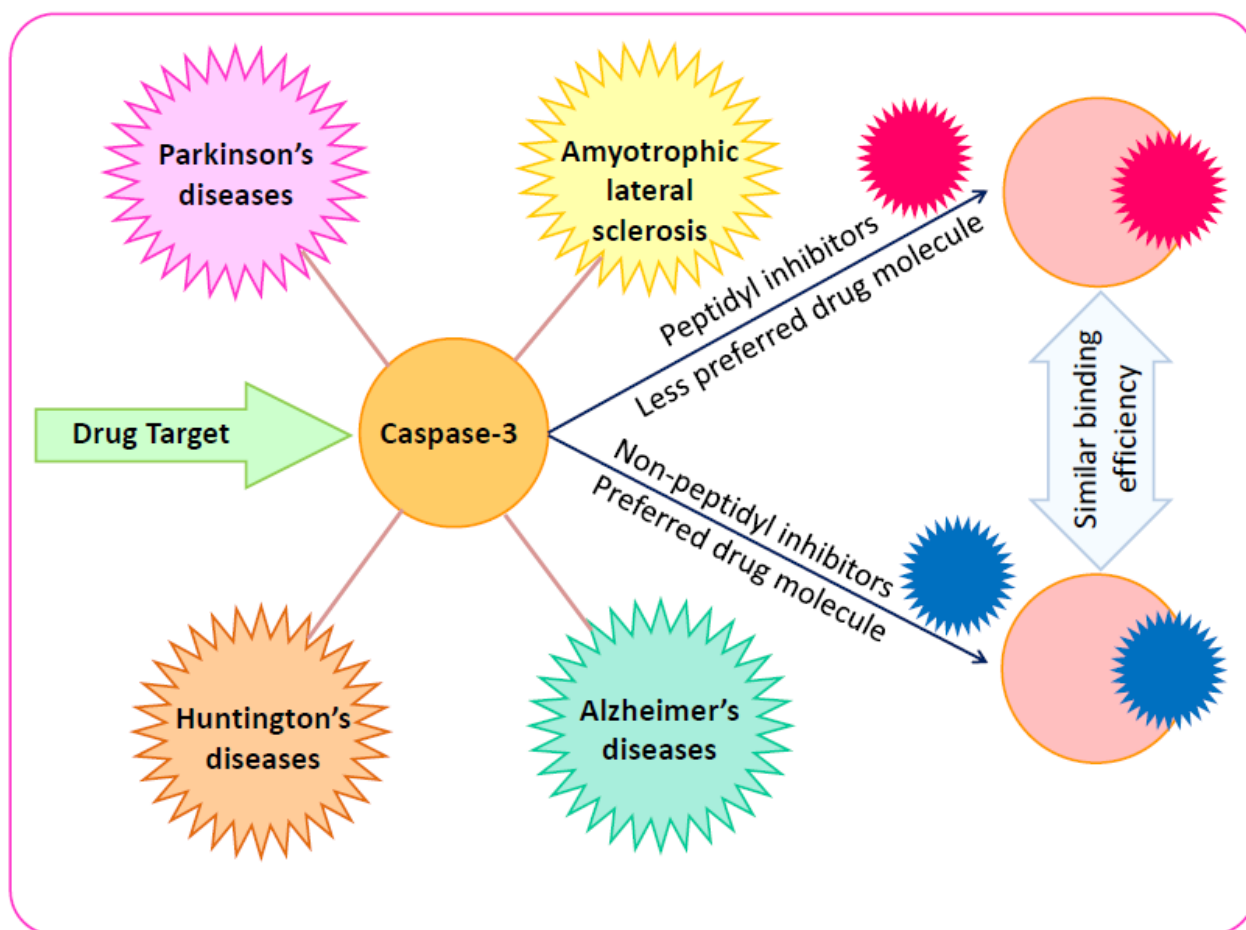

Supplement: Supplementary file 1 — Caspase 3 has been targeted by several researchers to combat multi-neurodegenerative (viz., Alzheimer's disease, Parkinson's disease, Huntington's diseases, and Amyotrophic lateral sclerosis) disorders. Several peptidyl inhibitors of caspase-3 have been identified. Owing to inherent difficulty in the handling and drug development from peptides, peptidyl inhibitors are not the preferred leads. Peptide based drugs result in higher drug development and handling cost. Moreover the efficiency of peptide may be compromised if the storage environment somehow deviates from the prescribed conditions. We have identified potent natural non-peptidyl inhibitors of caspase-3, effectively mimicking the interactions and binding affinity of peptidyl inhibitors. [file 379817.f1.pdf]
